# Oral Health and Quality of Life in Acromegaly: A Questionnaire-Based Study

**DOI:** 10.3390/dj13060226

**Published:** 2025-05-22

**Authors:** Giovanni Bruno, Francesca Dassie, Giorgia Preo, Ayoub Boutarbouche, Sara Brandolese, Pietro Maffei, Patrizio Bollero, Antonio Gracco, Michele Basilicata, Alberto De Stefani

**Affiliations:** 1 Department of Neuroscience, University of Padua, 35121 Padua, Italyayoub.boutarbouche@studenti.unipd.it (A.B.); alberto.destefani@unipd.it (A.D.S.); 2 Department of Industrial Engineering, University of Rome Tor Vergata, 00133 Rome, Italy; 3 Department of Medicine (DIMED), University of Padua, 35121 Padua, Italy; 4 Clinica Medica 3, Azienda Ospedaliera Università di Padova, 35121 Padua, Italy; 5 UOSD Special Care Dentistry, Department of System Medicine, University of Roma Tor Vergata, 00133 Rome, Italy; 6 UOSD Special Care Dentistry, Department of Experimental Medicine and Surgery, University of Roma Tor Vergata, 00133 Rome, Italy; michele.basilicata@ptvonline.it; 7 Department of Pharmacological Sciences, University of Padua, 35121 Padua, Italy

**Keywords:** acromegaly, Epworth Sleepiness Scale, Oral Health Impact Profile-14, OHIP-14, quality of life, ACROQOL

## Abstract

**Background/Objectives**: Acromegaly is a rare chronic disease caused by excess growth hormone (GH) and insulin-like growth hormone 1 (IGF-1) due to a pituitary adenoma. In acromegaly patients, oral and facial manifestations, such as mandibular growth, macroglossia, and dental malocclusion, are common and can affect quality of life. The aims of the present study were to evaluate the diagnostic path of these patients, the impact that acromegaly had on their oral health, the medical figures involved, and the role played by their dentist. **Methods**: The data were collected via an anonymous questionnaire to study dental health, dental care, and acromegaly diagnosis and history and via validated questionnaires. The validated questionnaires used were the ESS (Epworth Sleepiness Scale) to assess daytime sleepiness, OHIP-14 (Oral Health Impact Profile-14) to study perceptions of oral health, and AcroQoL to explore quality of life. **Results**: We enrolled 90 acromegaly patients: 48% of the patients reported acromegaly oral manifestations and 73% reported facial changes. The most frequent oro-facial manifestations reported by the patients were jaw growth (41%), diastema (40%), macroglossia (39%), and increased size of cheekbones (35%). The median OHIP-14 value was 5 (min 0–max 43), and the highest values were recorded in the questions relating to pronunciation difficulties and problems eating due to dental problems, as well as discomfort with dental aesthetics. The patients’ sleep quality was rated as good by 33% of patients, decent by 47%, and bad by 20%. The median ACROQol score achieved by the patients was 69 (min 19, max 98). An inverse and statistically significant correlation was observed between OHIP-14 and AcroQoL scores (Spearman correlation coefficient—0.44, *p* = 0.0002). **Conclusions**: Oro-facial changes significantly affect quality of life in cases of acromegaly, yet dental professionals’ involvement in diagnosis and management is limited. Greater awareness among and integration of dental professionals could support earlier detection and improve patient outcomes.

## 1. Introduction

Acromegaly is a rare chronic disease characterized by excessive growth hormone (GH) secretion due to a pituitary adenoma. It causes increased circulating levels of insulin-like growth factor 1 (IGF-1), causing physical alterations, somatic changes, and systemic comorbidities such as cardiovascular, respiratory, and osteoarticular manifestations [1,2,3,4,5,6,7,8,9,10]. The most common and early manifestations of acromegaly are facial changes, oral–dental signs, enlargement of the extremities, and thickening of the soft tissues [11,12,13,14,15,16]. The disease has a prevalence that varies from 20 to 130 people per million inhabitants [2,4,7,8,9,10,11,12,13,14,15], with an annual incidence of 2 to 11 cases per million people [4,7,8,9,10,11,12,13,14,15]. The average age at the time of diagnosis varies from 40 to 50 years [2,6,7,8,9,10,11,12], with younger patients tending to have a faster evolution of the disease [16].

The oral and facial manifestations are among the most common signs of acromegaly, being the third most frequent alteration at diagnosis, immediately after an increase in the size of the feet and hands. Some studies in the literature revealed that approximately 80% of patients with acromegaly have oral–dental–facial disorders, in particular macroglossia (54–58%), dental diastemas (40–43%), an increase in jaw size over time (22–24%), and mandibular prognathism (20–22%). Other maxillofacial manifestations of GH excess include hypertrophy of the submandibular glands, hypertrophy of the paranasal sinuses, and pain in the maxillofacial area [17,18,19,20]. The quality of life (QoL) of patients with acromegaly is often significantly impaired due to the physical, psychological, and social consequences of the disease. These include visible facial changes, chronic pain, fatigue, and functional limitations, all of which can affect self-esteem, interpersonal relationships, and daily functioning. The Acromegaly Quality of Life Questionnaire (AcroQoL) is commonly used to evaluate the impact of the disease on both physical and psychological dimensions. Among the comorbidities contributing to reduced QoL, obstructive sleep apnea syndrome (OSAS) plays a major role. OSAS is highly prevalent in acromegaly and is characterized by repeated episodes of upper airway obstruction during sleep, often caused by soft tissue hypertrophy and craniofacial abnormalities such as macroglossia and mandibular prognathism [21,22,23,24]. Radiographic examinations may reveal cephalometric alterations, such as increased mandibular dimensions and skull bases. These oral and facial changes might be present up to 10 years before diagnosis. Early identification of these manifestations could lead to early diagnosis and improve the prognosis and quality of life of affected patients [17]. Dentists, despite their privileged clinical position, are not currently significantly involved in the diagnosis of acromegaly [25,26,27]. Orthodontists, for example, might use dental photographs and X-rays to spot signs of disease; artificial intelligence could be used to assist in early diagnosis. Additionally, dentists could also help diagnose comorbidities associated with acromegaly, such as obstructive sleep apnea syndrome [28,29,30].

Although oro-facial manifestations are common and dental professionals routinely use photographs and radiographs in clinical practice, dentists and orthodontists are still not fully involved in the early diagnosis of acromegaly. This is often due to limited awareness of the disease and a lack of specific training in recognizing its early signs. Enhancing education on acromegaly and including targeted content in dental curricula and continuing education programs could strengthen the diagnostic role of dentists. A more attentive and integrated approach would also encourage collaboration between specialists, leading to earlier diagnosis and better outcomes for patients.

The aim of this study was to assess the diagnostic path of acromegalic patients, the impact of the disease on their oral health, the involvement of healthcare professionals, and patients’ perceptions of their oral and psychosocial well-being. Additionally, the study explored the relationship between dentofacial manifestations, quality of life, sleep quality, and patient awareness of these issues. The null hypothesis of this study is that there is no significant association between acromegaly-related oro-dentofacial manifestations and the patient’s quality of life, oral health status, or psychosocial well-being.

## 2. Materials and Methods

This cross-sectional observational study was conducted in collaboration with Clinica Medica 3 at the Medicine Department of Padua Hospital between January 2023 and December 2024. The study was approved by the Ethical Committee of Azienda Ospedaliera di Padova (CET-ACEV: 6189/AO/25; approved on 23 January 2025). The inclusion criteria were as follows: confirmed diagnosis of acromegaly, age ≥ 18 years, and ability to provide informed consent and complete the questionnaire. The exclusion criteria were severe cognitive impairment and language barriers precluding questionnaire completion. An anonymous questionnaire was created which would allow us to investigate the existence of a relationship between oral health, the oro-facial signs of acromegaly, the general progress of the pathology, and the impact that the disease and its oral manifestations have on facial effects have on the patient’s psycho-physical well-being. Patients were asked to indicate their age and the time interval in which the diagnosis of acromegaly was made. In the second section of the questionnaire addressed to patients, the interviewee’s relationship with their dentist was analyzed, and an essential dental history was taken with a focus on the presence of prostheses and/or implants and in what state they were. The process that led to the diagnostic confirmation of acromegaly was then investigated, asking the patient what the signs and symptoms were that led to the diagnosis and who the medical figure was who had a central role in this process. Finally, the perception of the patient’s sleep quality and the role that the dentist can have in sleep medicine was evaluated. The main variables collected were sociodemographic data (age, sex, age at diagnosis), dental history (presence and status of prostheses or implants, frequency of dental visits), oro-facial symptoms (jaw growth, diastemas, macroglossia, occlusal changes), diagnostic pathway (signs/symptoms leading to diagnosis and the medical professional involved), perceived sleep quality, and awareness of the dentist’s role in sleep medicine.

Three questionnaires validated in the literature were also used in their Italian forms [31,32,33]:

- Oral Health Impact Profile-14 (OHIP-14), a questionnaire that investigates the perception that patients have of their oral health status and the problems connected to it. To each of the 14 questions, the patient can respond with values on a scale from 0 (never) to 4 (very often/every day); the higher the patient’s total score, the worse their quality of life in relation to oral health (min score 0, max score 56).
- The Epworth Sleepiness Scale (ESS), a test for the subjective evaluation of daytime sleepiness, routinely used by dentists dealing with sleep disorders. For each situation proposed in the questionnaire, the patient is required to give a score from 0 (no possibility of falling asleep) to 3 (high probability of falling asleep). The scores obtained are then added and give a resulting value from 0 to 24 points. Scores up to 6 can be considered physiological, while scores above 7 indicate an increasing degree of daytime sleepiness.
- AcroQoL, a questionnaire designed for use in clinical trials and for routine monitoring of patients diagnosed with acromegaly. The questionnaire contains 22 items divided into two scales: one that evaluates the physical aspects and another that evaluates the psychological aspects. For each of the 22 questions, the patient is required to give a score from 1 (always) to 5 (never) based on the probability that each of the proposed situations occurs. The patient’s total score is calculated with the formula [(x) − 22/(110 − 22)] × 100.

In the first phase of this study, the form, in a reduced form, was sent electronically to patients intercepted in the department; some contacts were also reported by the association ANIPI Italia (Italian National Association of Pituitary Pathologies) and the ANIPI local section for the Veneto region. There were 26 questionnaires collected using this method. The questionnaire was then integrated with an AcroQoL section, and the final version was delivered to the patients in person, with the collaboration of the healthcare staff of the Medical Clinic department of Padua Hospital. The responses collected through this method numbered 64. In total, the questionnaires completed by patients and the subjects of this study numbered 90.

### Statistical Analysis of the Collected Data

Categorical data are expressed as frequencies and percentages, while numerical data are expressed as medians and interquartile ranges (IQRs). Categorical data were compared between groups with the Fisher test and Chi Square test, while numerical data were compared between groups with the Mann–Whitney test and the Kruskal–Wallis test. Correlations between numerical variables were assessed with the Spearman correlation coefficient. All tests were 2-sided, and a *p*-value less than 0.05 was considered statistically significant. The data analysis was carried out with R 4.1 (R Foundation for Statistical Computing, Vienna, Austria) REF.

## 3. Results

### 3.1. Description of the Sample

This study included 90 acromegalic patients (44 women and 46 men). The most represented age groups were those aged 31 to 59 (47% of those interviewed) and those over 60 (47% of those interviewed). Most patients were diagnosed between the ages of 31 and 59 (64%). No statistically significant difference was found between women and men regarding age at the time of the interview (*p* = 0.32) or age at diagnosis (*p* = 0.55).

### 3.2. Dental Manifestations and Care

Of those interviewed, 66% said they attend periodic check-ups at the dentist, but only a small percentage of these reported having their face and oral cavity photographed systematically by their dentists (13–26%). Instead, women declared that they attend dental follow-ups more frequently than men (women vs. men: 77% vs. 56%, *p* = 0.04).

The majority of patients said they have noticed both oral (48%) and facial (73%) manifestations as a result of acromegaly; no statistically significant difference was found between women and men regarding both oral and facial manifestations. Only one-third of patients have discussed these changes with their dentists, and men reported dental changes to their dentist more often than women (women vs. men: 21% vs. 41%, *p* = 0.04). The most frequently noted changes are described in Table 1. There was no statistically significant difference between women and men in whether they noticed facial (*p* = 0.78) or oral (*p* = 0.99) changes due to acromegaly. Most patients (77%) did not report problems with the temporomandibular joint (TMJ). Dentists were involved in acromegaly patient follow-up in cases of headache (13% of patients), jaw joint problems (6%), and facial pain (2%) (Table 1).

Approximately half of the interviewees wear a mobile or fixed prosthesis in their oral cavity. Of these prosthesis wearers, 44% report having had, or still having, problems with the devices. Patients who said they had implants (44% of those interviewed) stated the presence of problems less frequently, with almost identical incidences of onset before (6%) and after (8%) the diagnosis of acromegaly. Orthognathic surgery, to correct prognathism arising due to acromegaly, was proposed by a dentist to only a small percentage of interviewees (13%).

The symptoms/signs that most frequently led patients to their diagnosis were the presence of headache (reported by 29% of patients), joint pain (37%), alterations in the somatic characteristics of the face and general physical characteristics (37% for both), and the presence of apnea/snoring (26%) The medical figure who was most often involved in the diagnostic process was an endocrinologist (52%). A dentist was reported to be involved in the diagnostic process in only 11% of cases. A statistically significant association was found between the fact that a dentist was involved in the diagnosis and the fact that they took photos of the face (*p* = 0.003) but not of the oral cavity (*p* = 0.44).

Regarding treatment effects, only 20% of cases reported having decreased in size after the start of therapy for acromegaly, and 42% of patients reported no changes in tongue size after treatment.

### 3.3. Oral Health Impact Profile-14 (OHIP-14)

The results of the OHIP-14 questionnaire are described in Table 2. The median OHIP-14 value achieved by the interviewees was 5, and the highest values were recorded in the questions relating to pronunciation difficulties and problems with eating due to dental problems, as well as discomfort with dental aesthetics.

As regards perceived changes, a greater OHIP-14 score was observed in those who perceived alterations in occlusion (*p* = 0.005), spaces between teeth (*p* = 0.006), denture problems (*p* = 0.01), problems with chewing or speaking (*p* = 0.005), and increased tongue size (*p* = 0.003).

### 3.4. Sleep Disorders and ESS

The patients’ sleep quality was rated as good by 33% of patients, decent by 47%, and bad by 20%. In all, 9% of patients reported sleep apnea, 30% snoring, and 16% both apnea and snoring. The median ESS value achieved by the patients interviewed was 5, but more than one-third of the patients recorded a daytime drowsiness value greater than or equal to 7, a value which suggests a level of drowsiness higher than normal. The median Epworth Sleepiness Scale score was 5 (IQR 3–7) for subjects who reported good sleep quality, 5 (IQR 3–7) for subjects who reported fair sleep quality, and 8 (IQR 3–11) for subjects who reported poor sleep quality (*p* = 0.15; not statistically significant).

Despite this, only 30% of patients said they see a doctor for sleep problems. About 80% of patients were not aware that their dentist can also have a role in sleep medicine.

### 3.5. Quality of Life

The median ACROQol score achieved by the patients was 69 (IQR 60–82; min 19, max 98). The statements that proved to have the most impact on patients’ lives were those regarding physical appearance, the presence of joint pain, the conditioning of life due to physical changes, and the fact of feeling like a sick person. ACROQoL was worse in female patients compare to male patients (median for women vs. median for men: 64 (IQR 42–79) vs. 75 (IQR 67–83); *p* = 0.008).

The only oral or facial change associated with AcroQoL was facial skin change: patients who reported noticing a facial skin change had a lower AcroQoL score than patients who did not report noticing a facial skin change (median for patients with facial skin change vs. median for patients without facial skin change: 63 (IQR 35–74) vs. 72 (IQR 61–82); *p* = 0.02). The data are shown in Table 3.

The associations (separated by sex) between perceived oral and facial changes and quality of life (AcroQoL) are shown in Table 4. In men, reported joint pain was associated with a lower quality of life (measured by AcroQoL) (*p* = 0.02). In women, reported snoring/apnea was associated with a lower quality of life (measured by AcroQoL) (*p* = 0.01).

An inverse and statistically significant correlation was observed between OHIP-14 and AcroQoL scores (Spearman correlation coefficient—0.44, *p* = 0.0002). Patients who reported greater negative impacts on their oral health (higher OHIP-14 scores) tended to have worse overall quality of life (lower AcroQoL scores). Women reported a higher OHIP-14 score (*p* = 0.02) and a lower AcroQoL score (*p* = 0.008) compared to men, while no statistically significant association was observed between OHIP-14 or AcroQoL and age.

## 4. Discussion

The present study confirms that oro-facial changes in acromegaly are frequent complications and are difficult to reverse with treatment after GH excess. Despite this, only a portion of patients undergo regular dental follow-ups, and a small percentage of patients are offered orthognathic surgery. The dental manifestations secondary to acromegaly that most affect the perception of dental health include malocclusion, diastema, chewing and swallowing issues, dental problems, and macroglossia. These issues negatively impact the patient’s quality of life, as reflected in the AcroQoL questionnaire.

Several studies in the literature [30,34,35,36,37] have revealed that approximately 80% of acromegaly patients exhibit oro-dentofacial disorders, particularly macroglossia (54–58%), dental diastemas (40–43%), mandibular growth (22–24%), and mandibular prognathism (20–22%). These signs were present at diagnosis in most cases and often preceded the diagnosis by up to 10 years. Notably, facial changes are among the most common signs of acromegaly, ranking as the third most frequent alteration at diagnosis, immediately following increases in the size of the feet and hands. Our study results align with these findings: 48% of patients reported noticing specific oral cavity manifestations, and 73% reported pathological facial changes. However, unlike previous reports, our study found that the most frequently perceived changes by patients, in decreasing order of importance, were jaw enlargement, the appearance of diastemas, macroglossia, increased projection of the cheekbones and forehead, and dental occlusion alterations [38,39,40,41]. The majority of patients did not report problems affecting the temporomandibular joint, despite the literature reporting changes in the disease at this level as well [17,42,43].

Most of the literature on the subject suggests that, alongside rheumatic pain and neuropathies, oro-facial changes are evident and represent one of the main determinants of acromegaly patients’ quality of life [34,35,36,37,38,39,40,41]. In this study, an inverse and statistically significant correlation was found between the impact of the pathology at the oro-facial level (OHIP-14 questionnaire) and the quality of life perceived by the patients (AcroQoL questionnaire). Statistically, the only sign reported by patients that was significantly associated with the AcroQoL outcome was facial skin changes: patients who reported noticing a facial skin change had a lower AcroQoL score than those patients who did not. The age and gender of the patients in this study were not significantly associated with AcroQoL scores. This appears to be in agreement with most of the literature, even if there is no unanimity on the topic [34,35,36,37,38,39,40,41]. Another study [17] similarly demonstrated using the Short-Form 36 (SF-36) Health Survey questionnaire that experiencing oral signs and facial symptoms resulted in a worse score, indicating a worse quality of life, compared to subjects who did not present oro-facial signs or symptoms. These data suggest that early diagnosis of acromegaly could benefit patients both in terms of physical health and in terms of psychosocial well-being and quality of life [17,24,26].

In the present study, among the oro-facial manifestations that affected the OHIP-14 score and QoL were speaking or chewing difficulties due to an increase in the size of the tongue and malocclusion, impaired feeding processes secondary to oral cavity pain, tooth or prosthesis problems, and discomfort secondary to non-optimal dental aesthetics because of interdental diastemas. Accordingly, in the literature, a significant number of patients reported having problems with prosthetic aids in their oral cavity [17,44,45,46]. The impaired perception of dental health among acromegaly patients was associated with decreased quality of life [34]. The statements most frequently shared by the patients interviewed were related to their physical appearance (e.g., “I look horrible in photos”, “I see myself differently in the mirror”, “I have body parts that are too large”), joint pain, the impact of physical changes on their life, and the psychological belief of being a “sick person.” Even though the oro-facial and dental manifestations of acromegaly are perceived as significant by both patients and clinicians, there are no suggestions for treatment or monitoring of these comorbidities in the guidelines for the management and treatment of acromegaly. Dentists and orthodontists [30] may have a potentially significant role in identifying acromegaly patients, not only by noting oro-facial manifestations but also as sleep disorder experts. Oral and facial manifestations span between 48 and 73%, and sleep disorders are present in 9–30% of patients, but only one-third have discussed these changes with their dentist. Numerous studies, despite the encouraging results given by patient management in collaboration with a dentist and a maxillofacial surgeon, agree in stating that the role of this medical figure is still marginal within multidisciplinary teams for the management of acromegalic patients [26,27,30,47,48,49]. From this study, it emerged that, despite the possibility of them identifying and documenting early signs of disease, dentists are rarely involved in the diagnostic process of acromegaly (only 11% of cases). However, a statistically significant association emerged between the fact that a dentist was involved in the diagnosis and the fact that they collected photographic documentation of the face, indicating that greater awareness on the part of the professional can lead them to take on a significant role in making an early diagnosis. Of those interviewed, 66% said they attend periodic check-ups at the dentist, but only a small percentage of these reported having their face and oral cavity photographed systematically by their trusted dentist. In this sense, orthodontists could play a significant role [30].

Another role of the dentist could be to identify patients who present OSAS. Interestingly, one-third of patients listed snoring/apnea among the symptoms that led to their diagnosis [6,9,50,51,52]. Sleep disorders are associated with poor neurocognitive performance and a series of daytime symptoms, such as drowsiness, fatigue, and poor concentration. These determine a substantial worsening of quality of life, as well as a worsening of the systemic picture of the acromegalic patient, predisposing them to cardiovascular and metabolic pathologies [53]. According to our study, reported snoring and/or apnea in women was associated with a lower quality of life. The data from our questionnaire in relation to the quality of patients’ sleep could be an indication of their lack of awareness of the problem: even though most of the patients said they suffered from snoring or sleep apnea, they considered the quality of their sleep to be fair.

One of the main strengths of this study is the integration of both subjective patient-reported outcomes and clinical observations, offering a comprehensive understanding of how oro-facial changes affect quality of life in individuals with acromegaly. The use of validated questionnaires such as AcroQoL and OHIP-14 adds robustness to the data analysis and enhances comparability with other studies. Additionally, this study explored a relatively under-investigated area—the role of dental professionals in the diagnostic and therapeutic pathway of acromegaly—highlighting a critical gap in current multidisciplinary management. However, some limitations should be acknowledged. The study sample was composed of self-reported responses, which may have introduced recall bias and subjective interpretations of symptoms. Moreover, while the sample size was adequate, it was limited to a specific geographic context, potentially affecting the generalizability of the results. Another limitation is the cross-sectional nature of the study, which does not allow for establishing causal relationships. Lastly, while this study emphasized the potential role of dentists and orthodontists, it did not directly evaluate the knowledge or diagnostic accuracy of these professionals through clinical tests, which could be an area for future research.

## 5. Conclusions

This study demonstrates the significant impact of oro-facial manifestations of acromegaly on patients’ quality of life and underscores the underrecognized role of dental professionals in disease management.

Oro-facial changes are frequent and impactful, with macroglossia, diastemas, and jaw enlargement contributing notably to reduced quality of life, especially when facial skin changes are perceived by the patient. Dentists and orthodontists are underutilized in the diagnostic process, despite their regular clinical exposure to facial and oral structures and their potential to detect early signs of acromegaly.

Regular dental follow-ups are common, but few patients receive photographic documentation that could support earlier recognition of progressive facial and oral alterations. Integrating dental professionals into multidisciplinary care, particularly in screening for sleep disorders and managing prosthetic complications, could improve both early diagnosis and long-term quality of life for patients.

## Figures and Tables

**Table 1:** Acromegaly patients reporting oral and facial changes (Chi Square test and Fisher test).

Study Sample 90 (44 Women and 46 Men)	
Oral and Facial Changes	N (%)
Alterations to dental occlusion	26 (29)
Jaw growth	37 (41)
Spaces between teeth	36 (40)
Prosthetic problems	12 (13)
Trouble chewing/talking	19 (21)
Increased tongue size	35 (39)
Changes in the mucous membrane of the oral cavity	11 (12)
Lip alterations	21 (23)
Changes in the skin of the face	19 (21)
Increased forehead size	26 (29)
Increased size of cheekbones	31 (34)
Facial pain	12 (13)

**Table 2:** Results related to the impact of oral health in acromegaly patients, obtained using the Oral Health Impact Profile-14 questionnaire (Mann–Whitney test).

Proposed Situation	Answer	N (%)
Had trouble pronouncing words due to problems with teeth, mouth, or dentures	Never Hardly ever Sometimes Quite often Very often	47 (52) 18 (20) 19 (21) 6 (7) 0 (0)
Did you find that your sense of taste had worsened due to problems with your teeth, mouth, or dentures?	Never Hardly ever Sometimes Quite often Very often	60 (67) 14 (16) 12 (13) 4 (4) 0 (0)
Did you have any nagging pains in your mouth?	Never Hardly ever Sometimes Quite often Very often	36 (40) 24 (27) 25 (28) 3 (3) 2 (2)
Did you find it difficult to eat certain foods due to problems with your teeth, mouth, or dentures?	Never Hardly ever Sometimes Quite often Very often	34 (38) 18 (20) 30 (33) 6 (7) 2 (2)
Did you feel uncomfortable in front of others because of problems with your teeth, mouth, or dentures?	Never Hardly ever Sometimes Quite often Very often	52 (58) 11 (12) 18 (20) 6 (7) 3 (3)
Did you feel tense or nervous because of problems with your teeth, mouth, or dentures?	Never Hardly ever Sometimes Quite often Very often	49 (54) 14 (16) 24 (27) 2 (2) 1 (1)
Was your diet unsatisfactory due to problems with your teeth, mouth, or dentures?	Never Hardly ever Sometimes Quite often Very often	63 (70) 13 (14) 12 (13) 2 (2) 0 (0)
Did you have to stop meals because of problems with your teeth, mouth, or dentures?	Never Hardly ever Sometimes Quite often Very often	63 (70) 16 (18) 11 (12) 0 (0) 0 (0)
Have you found it difficult to relax due to problems with your teeth, mouth, or dentures?	Never Hardly ever Sometimes Quite often Very often	58 (64) 19 (21) 12 (13) 1 (1) 0 (0)
Did you feel a little embarrassed because of problems with your teeth, mouth, or dentures?	Never Hardly ever Sometimes Quite often Very often	52 (58) 15 (17) 18 (20) 4 (4) 1 (1)
Did you feel a little irritable with other people because of your teeth, mouth, or dentures?	Never Hardly ever Sometimes Quite often Very often	59 (66) 14 (16) 14 (16) 1 (1) 2 (2)
Did you have difficulty doing the usual jobs due to problems with your teeth, mouth, or dentures?	Never Hardly ever Sometimes Quite often Very often	70 (78) 11 (12) 7 (8) 2 (2) 0 (0)
Did you find that life in general was less satisfying due to problems with teeth, mouth, or dentures?	Never Hardly ever Sometimes Quite often Very often	51 (57) 19 (21) 18 (20) 2 (2) 0 (0)
Was he totally unable to act or do things because of problems with his teeth, mouth, or dentures?	Never Hardly ever Sometimes Quite often Very often often	73 (81) 11 (12) 5 (6) 1 (1) 0 (0)
Total sum	Median (IQR) Min–max	5 (2–13) 0–43

**Table 3:** Statistical relationships between scores on Acroqol, OHIP-14, and individual items of both questionnaires (Mann–Whitney test).

Variable	OHIP		ACROQOL	
	Median (IQR)	*p*-Value	Median (IQR)	*p*-Value
Age:				
less than 30 31–59	7 (5–9) 4 (2–10)	0.62	82 (79–86) 67 (55–79)	0.20
over 60	8 (1–14)		72 (61–82)	
Sex:				
F	9 (3–18)	0.02	64 (42–79)	0.008
M	4 (1–9)		75 (67–83)	
Age at diagnosis:				
less than 30 31–59 over 60	4 (1–8) 7 (2–17) 11 (4–13)	0.28	79 (61–86) 69 (61–81) 62 (46–72)	0.10
Perceived changes:				
Alterations to occlusion:				
no	4 (1–10)	0.005	69 (61–81)	0.99
Yes	10 (5–23)		75 (42–82)	
Jaw growth:				
no	4 (2–13)	0.47	69 (55–80)	0.45
Yes	7 (3–13)		75 (63–82)	
Spaces between teeth:				
no	4 (2–9)	0.006	68 (61–81)	0.75
Yes	10 (3–20)		73 (56–82)	
Prosthesis problems:				
no	5 (2–11)	0.01	73 (61–82)	0.06
Yes	13 (9–20)		61 (50–68)	
Problems chewing/speaking:				
no	5 (1–10)	0.005	71 (61–81)	0.26
Yes	11 (5–22)		65 (26–82)	
Increase in tongue size:				
no	4 (1–10)	0.003	73 (64–82)	0.06
Yes	10 (4–19)		63 (40–82)	
Changes in the oral mucosa:				
no	5 (2–13)	0.51	69 (60–82)	0.89
Yes	7 (5–9)		73 (58–81)	
Lip changes:				
no	4 (2–13)	0.06	72 (61–82)	0.35
Yes	9 (5–13)		64 (48–81)	
Changes in the skin of the face				
no	5 (2–13)	0.11	72 (61–82)	0.02
Yes	8 (4–17)		63 (35–74)	
Increased forehead size:				
no	4 (1–13)	0.06	72 (61–81)	0.34
Yes	7 (5–18)		62 (46–82)	
Increased size of cheekbones:				
no	5 (2–13)	0.55	72 (60–81)	0.89
Yes	7 (2–15)		68 (61–82)	

**Table 4:** Associations (separated by sex) between perceived changes and quality of life (Acroqol) (Mann–Whitney test).

Variable	Women (n = 33)		Men (n = 30)	
	ACROQOL: Median (IQR)	*p*-Value	ACROQOL: Median (IQR)	*p*-Value
Perceived changes:				
Alterations to occlusion:				
no	65 (50–78)	0.24	73 (64–83)	0.32
Yes	42 (29–80)		81 (73–83)	
Jaw growth:				
no	63 (41–78)	0.70	73 (68–88)	0.80
Yes	64 (46–82)		76 (65–82)	
Spaces between teeth:				
no	65 (46–79)	0.37	72 (64–82)	0.30
Yes	61 (25–76)		77 (72 -86)	
Prosthesis problems:				
no	64 (46–80)	0.28	76 (68–83)	0.11
Yes	50 (36–63)		67 (63–68)	
Problems chewing/speaking:				
no	65 (50–78)	0.31	75 (65–84)	0.97
Yes	46 (21–82)		75 (72–78)	
Increased tongue size:				
no	68 (57–80)	0.14	75 (67–86)	0.94
Yes	61 (36–71)		71 (68–82)	
Changes in the oral mucosa:				
no	62 (42–78)	0.49	75 (67–83)	0.56
Yes	74 (56–82)		73 (58–80)	

## Data Availability

Data and materials available on request.
